# Increasing Obesity in Brazil: Predicting a New Peak of Cardiovascular Mortality

**DOI:** 10.1590/S1516-31802000000600001

**Published:** 2000-11-01

**Figure 1:**
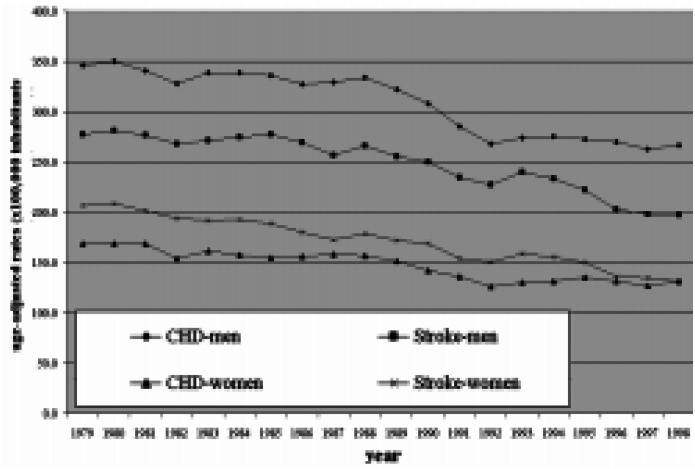
Age-adjusted mortality rates from CHD and Stroke for men and women aged 40 to 79 years in metropolitan areas of Brazil during the period from 1979 to 1998.

During the 1980s, cardiovascular mortality rates observed in Brazilian metropolitan areas were ranked among the highest in a comparison with certain selected countries.^1^ However, since then, these Brazilian rates have been decreasing with a peculiar pattern. During the 1990s, mortality rates from stroke followed a downwards trend, but mortality rates from coronary heart diseases (CHD) are now reaching a plateau without any evidence of a fall *(Figure 1)*.

**Figure 2:**
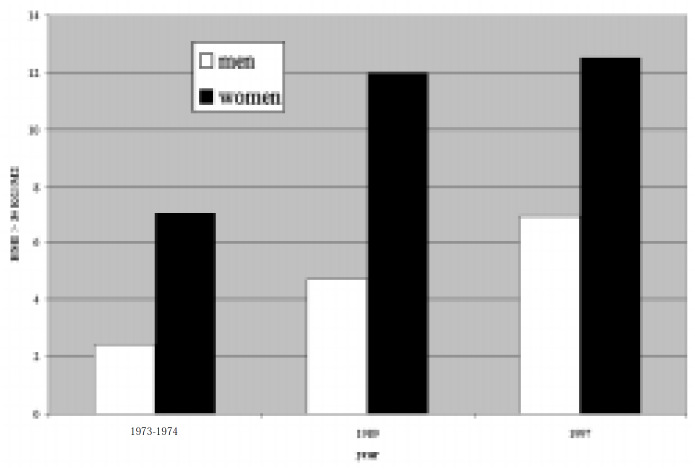
Obesity trends detected in three national cross-sectional studies in Brazil (reference #2).

On the other hand, three national cross-sectional studies performed in 1973-74, 1988 and 1996 showed an alarming and impressive increase in the prevalence of obesity, especially among city-dwellers.^2^ Over this period, the prevalence of obesity (body mass index greater than or equal to 30 kg/m^2^) increased from 2.4% (1973-74) to 6.9% (1996) among men, and from 7.0% (1973-74) to 12.5% among women *(Figure 2).*

The natural consequence of the obesity epidemic is an increase in the prevalence of diabetes mellitus, as proven in the United States, where diabetes prevalence rates jumped from 4.9% in 1990 to 6.5% in 1998 (+33%). This increase in diabetes prevalence accompanying the rise in obesity prevalence rates was observed in both sexes, all ages, all ethnic groups, and at all educational levels. The prevalence of diabetes was highly correlated with the prevalence of obesity among American states (r = 0.64, P < 0.001).^3^

Recently, one observational study has detected that the decline in CHD mortality deaths in the USA has been slower among patients with diabetes than among those without it.^4^ Other data from a cohort of American nurses has shown that the increase in body mass index explained an 8% increase in the incidence of CHD events.^5^

It is reasonable to speculate that the slowdown in the decline of CHD deaths in Brazil has been due to an increasing prevalence of both obesity and diabetes. Considering this fact, the epidemic of obesity must be halted, or else there will very probably be a new peak of coronary deaths in Brazil, in spite of the improvements regarding smoking restrictions and hypertension awareness and control that have been observed over the last few years.
